# Welfare, Work and the Conditions of Social Solidarity: British Campaigns to Defend Healthcare and Social Security

**DOI:** 10.1177/09500170211031454

**Published:** 2021-08-13

**Authors:** Genevieve Coderre-LaPalme, Ian Greer, Lisa Schulte

**Affiliations:** University of Birmingham, UK; Cornell University, USA; Middlesex University, UK

**Keywords:** mobilisation, organising, solidarity, unions, welfare states

## Abstract

When the welfare state is under attack from neoliberal reformers, how can trade unionists and other campaigners build solidarity to defend it? Based on 45 qualitative interviews, this article compares campaigns to defend British health services and social security benefits between 2007 and 2016. Building on the macro-insights of comparative welfare-state literature and the more micro-level insights of studies on mobilisation, community unionism and union strategy, it examines the factors that help or hinder the construction of solidarity. This research finds that building solidarity is more difficult when defending targeted benefits than universal ones, not only because of differences in public opinion and political support for services, but also because the labour process associated with targeting benefits, namely the assessing and sanctioning of clients, can generate conflicts among campaigners.

## Introduction

In recent decades, UK governments have sought to privatise and cut back the welfare state, leading to campaigns of resistance by trade unionists and other activists. With the Health and Social Care Act of 2012, for example, a conservative–liberal coalition sought to increase private-sector involvement in the National Health Service (NHS) by restructuring the commissioning of health services (Timmins, 2012). In 2007 and 2012, governments passed welfare reform acts to reduce the generosity of benefits, intensify controls over claimants and shift services into the private sector (Beck, 2018; Greer, 2016; Wiggan, 2012).

Campaigns of resistance require trade unionists and their allies to build and sustain solidarity. Framing matters in shaping solidarity, particularly when campaign discourse shifts away from employment issues (Foster and Scott, 1997). However, unions facing hostile governments may feel coerced into accepting privatisation or may compensate for lost influence through coalition building, member mobilisation and social justice framing (Teicher et al., 2006). Other studies have shown how even strong and militant unions underpinned by shared professional identification may fail in opposing privatisation (Kirton and Guillaume, 2019), and how engagement with policymakers and proposing alternatives can be a powerful tool to avoid it (Jalette and Hebdon, 2012).

The active building of solidarity depends on national institutions (Bolton and Laaser 2020; Morgan and Pulignano, 2020), especially in welfare services where institutions are infused with notions of social justice (Beck and Brook, 2020). Nonetheless, different parts of a country’s welfare state can be institutionalised and collectively understood in different ways (Bambra, 2005). The NHS is widely considered a ‘national treasure’, and public support for it has been consistently high since the mid-1980s; by contrast, British public opinion is divided over support for the unemployed. Comparative welfare scholarship provides a macro-sociological explanation for this difference: universal benefits engender cross-class political support, while targeted benefits stigmatise claimants and divide the working class (Korpi, 1980). In the welfare state, this has implications for worker mobilisation (Kelly, 1998), union strategy (Lévesque and Murray, 2010) and community-based organising (Holgate, 2018).

The aim of this article is to examine the factors that help or hinder the construction of solidarity in the defence of the welfare state. The core proposition is that *building solidarity should be more difficult when defending targeted benefits than universal ones.* The section that follows introduces neoliberal reforms in Britain’s healthcare and social security, two cases where trade unionists and activists mobilised to defend services. The proposition for this research is then grounded in the literatures on trade unionism (which focuses on active, micro-level building of solidarity) and welfare states (which concerns macro-level institutions and policy). The research design and the two cases are then discussed. Finally, a comparative discussion and conclusion is then presented with broader implications for the institutional factors that shape the building of solidarity.

## Neoliberal reforms in healthcare and social security

The NHS is a paradigmatic ‘national health system’ with state domination of funding and regulation, with near-universal public services free at the point of provision (Böhm et al., 2013). UK governments have a long history of privatising the NHS, mainly auxiliary services such as cleaning and catering, and to a lesser extent in clinical services. A key reform was the Conservative government’s 1990 NHS and Community Care Act, which introduced an ‘internal market’ for healthcare provision with a split between purchasers of care (regional health authorities) and providers (such as hospitals) (Timmins, 2012). Building on this, the Health and Social Care Act of 2012 (HSCA 2012) transferred purchasing responsibilities to 211 newly created local Clinical Commissioning Groups (CCGs), where ‘any qualified provider’, including private providers, could supply publicly funded healthcare (Davies, 2013).

Although privatisation was intended to reduce costs, cuts had a mixed effect on commissioning in healthcare. This is because austerity can stifle private-sector involvement if there is a squeeze on prices (and therefore profits) or a shift of risks onto private providers (Krachler and Greer, 2015; Robertson et al., 2017). Consequently, NHS provision has remained predominately public: only around 7–8% of spending in England goes to private providers (Department of Health, 2019) and only 11% of the population having supplementary private insurance to cover elective and more rapid and convenient access to care (Mossialos et al., 2018).

UK social security, by contrast, is the paradigmatic example of a ‘liberal welfare regime’ restricted in both scope and generosity (Esping-Andersen, 1990). In the 1980s, policymakers began to introduce work requirements and back them up with financial penalties known as sanctions, and in the 1990s they began systematically to encourage for-profit companies to deliver ever-larger packages of work (Greer et al., 2017).

With the Welfare Reform Act of 2007, the Labour government sought to expand the pool of claimants subject to work requirements. It abolished Incapacity Benefits, reassessed claimants, and created the Employment Support Allowance that included work requirements for disabled claimants. After 2010, the Conservative-led coalition government introduced a mandatory work-for-benefits (‘workfare’) scheme and a large, centralised, privatised welfare-to-work scheme, the Work Programme (Beck, 2018; Wiggan, 2012). In the Welfare Reform Act of 2012, the government further reduced the generosity of certain benefits and set in train the replacement of most benefits with the Universal Credit. Meanwhile, sanctioning escalated: the 580,000 sanctions levied in 2012–13 represented a 400% increase in just three years (PCS, 2014b). Tightened assessments of eligibility, extended work requirements and enforcement through sanctions were all methods for reducing benefit expenditures through intensified targeting.

Throughout this period of neoliberal reform, health professionals, Jobcentre staff, NHS campaigners and claimants groups fought back. Their grievances had similarities: cuts and privatisation degraded front-line jobs and weakened the benefits and services citizens depended on. Campaigners, however, faced different challenges in building solidarity, including different levels of public support for healthcare as opposed to unemployment benefits. Table 1 displays data from the International Social Science Programme showing that, since the 1980s, public support for government funding and provision of healthcare has been consistently strong, whereas public support for assisting the unemployed has declined. Consequently, healthcare and social security trade unions faced different conditions when building broad societal solidarity. The next section considers the factors that helped and hindered building solidarity in these two contexts.

**Table 1.** Respondents to ISSP in Great Britain: views on health and unemployment.

*It should be the government’s responsibility to* . . .										
	Provide healthcare for the sick					Provide a decent standard of living for the unemployed				
	1985	1990	1996	2006	2016	1985	1990	1996	2006	2016
Definitely should	0.854	0.850	0.817	0.732	0.682	0.443	0.320	0.288	0.134	0.105
Probably should	0.137	0.145	0.169	0.258	0.300	0.413	0.480	0.499	0.440	0.342
Probably not	0.006	0.004	0.011	0.007	0.014	0.108	0.151	0.147	0.314	0.356
Definitely not	0.003	0.001	0.003	0.003	0.004	0.037	0.048	0.066	0.113	0.197
*Government should spend on* . . .										
	Healthcare					Unemployment benefits				
	1985	1990	1996	2006	2016	1985	1990	1996	2006	2016
Much more	0.355	0.364	0.429	0.288	0.323	0.119	0.080	0.071	0.038	0.300
More	0.525	0.535	0.486	0.533	0.534	0.291	0.278	0.288	0.118	0.138
The same	0.112	0.096	0.081	0.164	0.136	0.397	0.468	0.440	0.458	0.434
Less	0.005	0.004	0.003	0.011	0.008	0.149	0.136	0.152	0.293	0.291
Much less	0.003	0.001	0.001	0.003	0.000	0.042	0.038	0.050	0.093	0.108

*Source*: International Social Survey Programme (ISSP), Role of Government module, various years.

## The construction of solidarity

Most sociologically minded writers on trade unionism emphasise the active role of workers constructing solidarity: ‘individuals knowing, engaging and potentially supporting other individuals around them’ (Beck and Brook, 2020: 8). The meaning of solidarity varies depending on shared characteristics, common interests pursued collectively, differences that coexist with mutuality, and who or what solidarity is against (Gumbrell-McCormick and Hyman, 2015). This literature often contrasts two faces of trade unionism: the ‘vested interests’ of members in jobs, income and working conditions, and serving as the ‘sword of justice’ fighting for goals not only of interest to members (Flanders, 1970). Solidarity is thus both exclusive and inclusive, with a varying definition of ‘us’ reinforced through bonding and bridging processes, and distinct from those considered as ‘them’ such as employers or other groups of workers (Morgan and Pulignano, 2020).

One strand of this literature considers how perceived injustice translates into collective action, including attribution, efficacy of the action, leadership of the group and opportunity structure (Kelly, 1998). Atzeni (2016) and López-Andreu (2020) have highlighted structural constraints and opportunities facing mobilising workers and the transformation of mobilisation into ‘spontaneous solidarity’ with allies. Tassinari and Maccarrone (2020), similarly, depict gig economy couriers able to shift embryonic solidarity (collective feelings of reciprocity and responsibility towards one another) into active solidarity through mobilisation.

A second strand examines union strategy, emphasising the organisational dimension of worker solidarity. Lévesque and Murray (2010), for example, distinguish ‘internal solidarity’ (a perception of a shared status and member participation in the union) from ‘external solidarity’ (work with other actors, such as civil society organisations or researchers). Gumbrell-McCormick and Hyman (2013) similarly refer to ‘organisational power’, which organises workers into a ‘community’, which will support the union’s goals and aims. Central issues are how unions can mobilise these resources (Ganz, 2000), combine them as part of a coherent organisational plan for revitalisation (Katz et al., 2003) and achieve a shared sense of political purpose to sustain solidarity (Upchurch et al., 2012).

A third strand, on coalition building and community-based organising, examines the extension of solidarity beyond the union and the work-group. Key issues include how exchanges between unions and other partners are characterised by trust and reciprocity (Wills and Simms, 2004), how unions can shift power relations (Tattersall, 2013), the conditions under which unions cooperate with others in civil society (Heery et al., 2012), and other ways in which workplace organisation can be anchored in working-class communities (McAlevey, 2016).

The above view of actively constructed worker solidarity contrasts with the approach of comparative welfare states scholars. While the former emphasises working-class solidarity and define it in terms of collective action, the latter emphasise cross-class solidarity and define solidarity as the willingness of social groups to give something up for the broader social welfare. Comparativists measure it not in terms of mobilisation and building worker power, but in terms of national-level policy outcomes (Baldwin, 1990) or public opinion (Banting and Kymlicka, 2017). They show how welfare-state architectures can generate, sustain, or corrode social solidarity. Korpi (1980: 305) argues that where benefits are targeted based on need, ‘the poverty line, in effect, splits the working class and tends to generate coalitions between the better-off workers and the middle class against the lower section of the working class’. Key findings are that universal welfare states are more effective at poverty and inequality reduction than targeted ones (Korpi and Palme, 1998) and that tightening work requirements through ‘workfare’ in the US has reinforced negative views of claimants and weaker public support for providing assistance (Soss and Schram, 2007). Universal welfare benefits therefore engender broader cross-class support and are less vulnerable to neoliberal reforms than targeted benefits.

In this macro-sociology, identities and interests are produced and reproduced by institutions; unions are exhorted to involve themselves in the policymaking process (Schelkle, 2011), but mobilisations of workers and communities play little role. Because of the institutionalised protections their members enjoy, unions are expected to form self-interested coalitions with employers opposed to the interests of precarious and unemployed workers (Emmenegger et al., 2012), and, because of their relative lack of influence and rights, unemployed workers’ movements may be radical but powerless (Giugni, 2009). Although this kind of analysis is conventionally used for international comparisons, Bambra (2005) notes that welfare politics vary domestically between service-intensive benefits (like healthcare) and cash benefits (like social security).

Some scholars have brought together these macro-institutional theories with more agent-centric accounts of worker mobilisation and the labour process. Doellgast et al. (2018) note that universalist welfare-state architectures are generally conducive to unions building broad and inclusive solidarity. Neoliberal reforms are an important part of the backdrop of deteriorating job quality and intensified management control in parts of Britain’s civil service (Carter et al., 2011) and US services for the unemployed (Esbenshade et al., 2016), a dynamic reinforced by market competition and private ownership (Greer et al., 2018). The ramping up of conditions and sanctions for the unemployed may contribute to precarious employment by forcing workers into insecure jobs and creating ways for them to cycle between work and unemployment (Greer, 2016). Privatisation can also threaten occupational norms, especially for workers whose clients are stigmatised, as Kirton and Guillaume (2019) show in their study of Britain’s probation service.

This article brings macro- and micro-level explanations together to examine the factors that help and hinder building solidarity in the defence of the welfare state. The starting point is the proposition that *building solidarity should be more difficult when defending targeted benefits than universal ones*. In keeping with the literature on unions, this should be reflected in campaigners’ efforts to build and sustain solidarity among themselves: they should have enough of a shared sense of purpose that conflicts do not spill over into the public sphere, poisoning relationships and preventing future work. In keeping with the welfare-state literature, this difference should be reflected in campaigners’ efforts to build broader societal solidarity: they should be able to mobilise public opinion and influence policy, or at least disrupt policy implementation. Below, the active construction of both kinds of solidarity is described.

## Methods

To examine this proposition, a structured comparison of two series of campaigns was used, chosen for variation in the outcomes of interest (level of difficulty in building solidarity) and possible conditions that could explain them (welfare state architectures and strategies and resources of campaigners) (Ragin, 2009). The first case is of unions and healthcare campaigners in England mobilising to stop the privatisation of NHS services following the introduction of the 2012 HSCA, with a focus on two local campaigns: Nuneaton and Bristol. The second is of unions and claimants groups mobilising to stop the rollback of social security benefits that started in 2007, national campaigns against the reassessments of disabled claimants, work-for-benefit schemes and sanctions. Building solidarity was generally more difficult for campaigners in social security than in healthcare, and this research was designed to understand why.

A total of 45 semi-structured interviews were conducted between 2007 and 2016, 42 of which were in-person and three over the phone. Roughly half were in London and the rest elsewhere in England and, for social security, Scotland (Table 2). Trade unionists and representatives of other groups who were involved at the organisational level or who had participated in mobilisation were selected. Four public forums with social security campaigners and several discussions of strategy also formed part of the data collection. Interviewees were identified at public events or through newsletters, social media, or recommendations from other interviewees; telephone or email were used to schedule interviews.

**Table 2.** Interviews and interviewees.

**Wave 1** 2007–2012 10 interviews	Social security	Unions	Unemployed Workers Centre: 2010 Amicus: 2007 PCS national: 1. 2008 2. 2010 3. 2011^a,b^ TUC: 1. 2007^b^ 2. 2007 UNISON: 1. 2007 2. 2008
		Non-unions	Claimants: 1. 2012^b^
**Wave 2** 2013–2016 35 interviews	Social security	Unions	PCS local: 1. 2013 2–6. 2014 7. 2015 PCS national 4. 2013 5. 2014^b^ 6. 2016^a,b^ Unite Community: 2013
		Non-unions	Claimants: 2. 2013 3. 2014^c^ 4. 2014^a^ Labour Party: 1. 2013^b^
	Healthcare	Unions	BMA 2014 RCN 2015 UNISON: 3. 2013 4. 2015^a,b^ 5. 2015 Unite: 1–2. 2013 3–4. 2013^a^ 5. 2016 6. 2016^b^
		Non-unions	Local health campaigners: 1–2. 2015 3–4. 2016^a^ 5. 2016 National health campaigners: 1–3. 2013 Labour Party: 2. 2015
Total: 45 interviews, 50 interviewees			

*Note*: ^a^repeat interviewee; ^b^two interviewees; ^c^three interviewees. BMA: British Medical Association; PCS: Public and Commercial Services Union; RCN: Royal College of Nursing; TUC: Trades Union Congress.

Interviews were recorded digitally, lasting between one and two hours. Participants were asked to describe how campaigning had unfolded, including the issues at stake, and the factors that contributed to and frustrated mobilisation. Ethical approval was obtained from the University Research Ethics Committee and participants’ written or verbal consent was obtained prior to the interviews.

The data include interview summaries and transcripts, field notes, blog posts and reports, and these were analysed with MaxQDA. The two narratives were then constructed using continuous comparisons between the two cases (Glaser and Strauss, 1967). The starting point of this analysis was the difference in the outcome; when the NHS was being studied, it was evident that building solidarity in social security had been more difficult. The key categories were derived subsequently through research team meetings, discussions after interviews or observations, open-ended (inductive) coding and detailed memos. When these stopped yielding new themes and instead corroborated existing insights, top-down (deductive) coding was used, in which the cases were categorised and quotes identified to illustrate concepts and evidence categorisations. The main codes were grouped as (1) solidarity among campaigners and (2) broader social solidarity, and this forms the structure of the next two sections.

## Healthcare

After the introduction of the HSCA 2012 and the subsequent decentralisation of commissioning services, campaigning mostly took place locally and was not nationally coordinated. Both campaigns reviewed started in 2013: in Nuneaton when regulators selected the hospital for a private takeover (Clover, 2013) and in Bristol when the CCG decided to put the city’s mental health services out to tender (Calkin, 2013). In both cases, private sector firms such as Circle, Serco, Priory Group and Care UK expressed interest in taking over services.

Private-sector involvement in these campaigns was viewed by some unions as a threat, leading to campaigns to stop privatisation. The main union involved was UNISON, which represented nurses, allied health professionals, porters, administrative staff and cleaners. In Bristol, the local UNISON branch mobilised alongside grassroots campaigning groups made up primarily of retired health professionals and patients. In Nuneaton, both UNISON and Unite branches were involved in campaigning against the private takeover of their local hospital. The fragmented landscape of NHS unions was a problem, not only because of the difficulty of coordinating the campaigns of the two largest unions, but also because occupational unions representing large numbers of workers, such as the Royal College of Nurses, remained neutral in both cases. Although consultation mechanisms were in place, meetings to decide on the restructuring of services were held in private, and unions were marginalised.

UNISON and Unite sought to protect members from the consequences of privatisation. They argued that pay, terms and conditions of employment were better in the NHS, and that privatisation led to worse jobs (UNISON, 2015; Unite, 2013). In Bristol, grassroots activists noted that staff were ‘terrified’ of being transferred to a private provider, saying ‘we must stay with [the public hospital] because otherwise it will be much worse’ (Local health campaigner 1). The threat of privatisation was also framed more broadly, as violating the universalistic principles of the NHS, with local campaigners viewing the HSCA as a step towards American-style healthcare. Local campaigners argued that profiteering would lead to cuts, poorer care and more stringent rationing. As one interviewee in Bristol noted: ‘We want to keep the NHS because it’s one of the jewels in the crown of this country; it’s what defines everything good about this country’ (Local health campaigner 5). The threat and injustices linked to healthcare privatisation prompted these local unions and grassroots campaigners to mobilise to protect NHS workers and public healthcare.

### Solidarity among campaigners

Interviewees in both cases reported difficulties in maintaining solidarity among campaigners, and unions did not always take the lead in these campaigns. Although NHS unions generally have a relatively high union density of 43% (Galetto et al., 2014), union interviewees found it difficult to involve branch members in campaigns. Reasons for this included ‘TUPE’ rules protecting workers transferred into privatised services, scepticism that privatisation would go ahead, vulnerability to employer reprisals and the physical exhaustion of workers (UNISON 5, Local health campaigner 6, Unite 5). As one grassroots campaigner in Bristol explained:

I think that hours that nurses work and the sort of weight of the work they carry with them means that they are time-strapped . . . There are ways in that the people who want to stand out and speak out are very easily labelled as troublemakers. You can be a union rep but the expectation is that you will not rock the boat. (Local health campaigner 6)

In both cases, local trade unionists lacked external solidarity networks at the campaign’s outset, and networks had to be built with other union branches, grassroots NHS campaign groups and local politicians. Despite common goals, campaigners reported some difficulty cooperating: they sometimes used different tactics and often criticised one another. In Nuneaton, UNISON and Unite branches campaigned separately against the takeover the hospital because of tactical disagreements: ‘Oh they hate me . . . I tried, I tried. [. . .] I met with the UNISON officers and whatever. And tried. And they just thought that our tactics were too heavy-handed’ (Unite 5). UNISON saw the duplication of campaigning as a positive development, arguing that this had overwhelmed NHS decision-makers: ‘Everyone came at [them] from a different angle’ (UNISON 6). Instead, Unite sought the support of the Labour Party candidates in the 2015 general elections, and working with Labour politicians became an important part of their campaign strategy:

‘. . . the fact that [politicians] were turning up gave us the leverage with the press, that was then getting the message out there with the public, and then the public were becoming more aware. (Unite 5)

In Bristol, UNISON worked primarily with a local grassroots NHS campaigning group, and the union branch ultimately did not lead the campaign. Some trade unionists attended campaign meetings but were reluctant to participate in protest action, fearing reprisals by their employer as previously mentioned (Local health campaigners 1 and 6). Some grassroots activists expressed disappointment with the lack of involvement by trade unionists and felt that they should be more involved in the ‘wider movement’ instead of focusing solely on jobs (Local health campaigner 1).

In both campaigns, unions and grassroots campaigners struggled to win support from doctors, and the Royal Colleges of Nursing and Midwives chose not to be involved. One grassroots campaigner in Bristol expressed frustration:

I think that the performance of the [Royal College] is hugely disappointing. I mean, where are they? . . . the official voice of [the profession] should come from the [Royal College] but they are virtually silent. [. . .] So, all this money goes into supporting them, and they do fuck all. (Local health campaigner 6)

Overall, solidarity among trade unionists and other local campaigners was variable; while unions were able to find allies, the NHS unions were fragmented and there was no national strategy. Nonetheless, difficulties and conflicts between local actors were hidden, with tactical and political disagreements on approach or tactics remaining out of the public arena.

### Broad social solidarity

Campaigners benefited from public support for the NHS and its principles which allowed for the successful mobilisation of both communities against privatisation initiatives. Campaigners used stalls and leafleting to both educate the public and themselves and to recruit activists. They also used petitions to attract media interest and build momentum. In Nuneaton, Unite explained:

The first couple of weeks, we were trying to entice people to come to the stall to sign our petition. By week 3 there was a queue. [. . .] They were taking petitions away with them and bringing them back on Saturday full up. Some would say, ‘I live in a block of flats, some of my friends can’t sign it so I’ll take it and come back’. It was absolutely amazing. (Unite 5)

Activists still faced some difficulties in convincing the public of the negative impact privatisation would have on NHS services. This included explaining the complexity of commissioning and the widespread view that privatisation would never happen (Local health campaigner 6). The association between privatisation and hotel-like private hospitals was also a problem, especially for the ‘aspirational working class’ (Local health campaigner 6): one campaigner in Bristol felt he was ‘too posh’ to communicate well with this group (Local health campaigner 1). A recent scandal in mid-Staffordshire highlighted dangerous conditions in parts of the NHS, and ‘those who were looking to denigrate the NHS would say look at this poor quality care that is being delivered here, we need to bring in more private sector’ (UNISON 6). Personal negative experiences with the NHS also mattered as those interviewed in Bristol noted:

a lot of people have had bad experiences. The NHS is not all wonderful. For the people who have seen their elderly folks uncomfortable and neglected in the hospital, the idea of the privatising can be seen as a good thing. (Local health campaigner 6)

Nevertheless, campaigners reported a widespread willingness to protect the NHS. As one campaigner with management experience told us: ‘in the NHS . . . people make a lot of noise and they just don’t do it (Labour Party 2). Those interviewed at UNISON described the emotional support for the NHS as ‘bound in this Britishness’: ‘This is the NHS. This is part of our being’ (UNISON 6). Those in Bristol evoked the memory of post-war Britain:

I think that view of the NHS has been formed by the people who knew what had been there before. So that . . . the post-war settlement of welfare state absolutely transformed the lives of working people . . . it brought such comfort. (Local health campaigner 6)

The successful ‘Save Lewisham Hospital’ campaign in 2013, which prevented the government from closing services, was noted by interviewees as a key moment in NHS campaigning; several other localities followed suit, convinced that they could protect their services. Following the 2015 general election, the government put several large commissioning initiatives on hold and began using euphemisms for privatisation: ‘externalisation, automation, and mutualisation . . . the rule is never using the p-word’ (UNISON 4).

The aim of the two campaigns examined was to disrupt privatisation initiatives, and both were successful in preventing a private-sector takeover of services. While campaigners with a common goal had difficulty developing joint campaigns, their efforts were facilitated by broad public support, which legitimised and bolstered their mobilisations to stop the privatisation of local healthcare services.

## Social security

In social security, campaigns were coordinated on a national scale. Between 2007 and 2010, the main actors were the Public and Commercial Services Union (PCS) (which claimed 80% membership in the government department that administers social security), the Trades Union Confederation (TUC) (which historically organised local Unemployed Workers Centres) and national charities representing lone parents and disabled people. Between 2010 and 2011, dozens of local claimants groups were established, as well the national activist networks Black Triangle, Disabled People Against the Cuts (DPAC) and Boycott Workfare. Because the civil service unions were not historically divided by occupation, PCS had a much stronger coordinating capacity than the NHS unions. In addition, Unite Community was created to organise unemployed and precarious workers, and the national charities retreated from campaigning in this area (TUC 1, PCS national 1, Unemployed Workers Centre).

The most immediate problems for campaigners – whether trade unionists or claimants – concerned cuts to benefits, assessments of claimants, work-for-benefits schemes and sanctions. PCS activists estimated that 40% of its members in the DWP had such low earnings that they would be eligible for the new Universal Credit benefit (PCS national 4) and found that sanctions were impinging on the labour process: 80% of members responding to one survey felt increased pressure to sanction claimants, and 20% had an actual numerical target (PCS, 2014b). PCS activists also bemoaned the lack of staff discretion in making assessments, arguing that the Work Capability Assessments for clients prescribed by the government were mechanical and divorced from common sense: ‘here’s a question, tick a box’ (PCS local 4).

For many claimants, activism was triggered by an encounter with the newly reformed benefits system. One group formed after a friend of the founders took his own life after being denied disability and housing benefits (Claimants 4). Another activist described being ‘rolled over’ from the old Incapacity Benefit onto the new Employment and Support Allowance, receiving ‘out of the blue’ a 21-page questionnaire to determine eligibility, being subjected to a traumatic in-person assessment at the private contractor Atos and having her application rejected, all leading to a deterioration of her mental health condition (Claimants 2).

### Solidarity among campaigners

Trade unions and claimants groups had divergent ways of organising. For PCS, organising, socialist politics and internal democracy were points of pride (Hodder, 2014; Upchurch et al., 2012) and Unite Community was an innovative organising project to make the union more inclusive (Holgate, 2018). While unions had national bureaucratic structures with offices and staff, claimants groups were organised locally and networked nationally using social media. They measured their resources in terms of social media followers and numbers of demonstrators (Claimants 1–4). Contact between unions and claimants groups were made quickly once the latter mobilised: ‘if people are protesting at the Jobcentre you get to know them!’ (PCS national 6).

Cooperation was the policy of the union leadership and the leaders of the claimants groups. One joint statement concluded: ‘PCS, DPAC, and Black Triangle members have a common cause in defeating these welfare cuts and in building a decent welfare state’ (Black Triangle, 2012). Cooperation included efforts to improve claimants’ access to information and publicise problems with cuts to benefits, with joint appearances at public events and claimants supporting the union in a pay dispute (PCS national 5). One interviewee argued that campaigners falling out with one another was a ‘betrayal’, and that it is the ‘unity in which it defends its members’ that is the ‘true measure of a movement’ (Claimants 4).

Cooperation proved difficult to sustain. One national PCS leader said that one of the main national networks was not involved in a campaign because it was not representative: ‘a man and his laptop’, this group lacked members and structure. Claimants groups were narrowly focused on particular problems, whereas PCS promoted a broader vision of universal government-provided social security (PCS, n.d.). Interviewees bemoaned conflicting objectives, narrowly defined interests and a lack of shared political vision (PCS national 4, Claimants 4).

Open conflict between PCS members and activist claimants broke out after a series of mobilisations at Atos offices against the reassessment of disabled claimants in 2011–2014. A PCS activist explained that relations with claimants groups were strained during this period: ‘They picket, call us Nazis, and . . . basically they’re not interested in anything that we have to say. If you say, “I’m just doing my job”, they say, “Where have I heard that before?”’ (PCS local 4). Although claimant activists recognised the pressures union members were under and apologised, two local union activists reported being angry with national officers for cooperating with claimants groups (PCS local 1 and 4). One complained that the union called on members to join protests at the offices of private contractors but did not do the same for public-sector Jobcentres (PCS local 4).

Claimants demanded that the union consider fighting sanctions through industrial action or ‘non-cooperation’. PCS’ 2013 annual conference passed a resolution to explore the possibility of including non-cooperation with sanctions in future industrial action ballots (PCS, 2014a). This did not satisfy the union’s critics. After an exchange of tweets with claimants groups, a top PCS official argued that ‘an instruction not to implement’ would be illegal, and the government ‘would have no hesitation in sacking people and therefore driving to replace a union organised workforce with a non-union workforce’ (The Socialist Party, 2013). In several furious responses, bloggers pointed to the suicides, destitution and homelessness that resulted and claimants groups’ past support for PCS members in industrial disputes.^1^

Campaigners’ shared grievances and their strategy of cooperation could have served as the basis for strong solidarity among them. However, open conflict broke out, triggered at the local level by union members’ roles in administering sanctions and assessing clients. The labour process of administering targeted benefits thus made solidarity difficult to build and sustain.

### Broad social solidarity

Campaigners reported difficulty in influencing policy and public opinion over social security and many times discussed a need to change the discourse in society. This problem was confirmed by a senior Labour Member of Parliament, who reiterated his support for sanctions and privatisation and did not name unions or claimants among the many sources from which he drew his ideas about social security policy (Labour Party 1). Even the TUC supported sanctions as part of Labour’s proposed job guarantee scheme:

Because they are real jobs, the same benefit rules that apply to other jobs should also apply; claimants who turn down a guaranteed job without good cause should face benefit sanctions. (TUC, 2013)

The cross-party consensus in favour of sanctions was a problem for campaigners, and part of PCS’ work was to convince the TUC to change its position (PCS, 2015). One trade unionist argued that for industrial action to be effective in fighting sanctions, the broader societal discourse would have to be different (strategy meeting January 2014), and another contrasted unemployment benefits with the universal state pensions and NHS, noting the difficulty of attracting public support to defend employment services:

You say to people, do you like your state pension? And people say ‘yes’. So, it depends, you have the deserving poor, and the undeserving poor. A lot is fed by the media, the negative portrayal of benefit claimants, TV shows . . . If you’re going to close a local hospital, it’s the kind of campaign where you’ll have a petition and a demo. But if you’re going to close a Jobcentre . . . it’s not instinctive public support. (PCS national 5)

For one claimant activist, countering popular narratives around unemployment benefits had been a top priority from the outset:

… just go on the website of the *Daily Mail*, or *The Sun*, and search the word ‘scrounger’, and you’ll come up with hundreds of articles . . . a demonisation of people who rely on benefits. We’ve exposed the racketeering of private companies in the creation of the disability assessment regime. (Claimants 4)

By organising, they sought to turn the tables on the welfare profiteers benefiting from the privatisation of welfare benefits administration and employment services.

These campaigns had some success in winning public support but did not lead to direct influence over public policy. PCS embarrassed the government with its survey on sanctions targets; the government denied knowledge of any targets and accused the PCS of being a ‘tool of the Labour party’ (meeting January 2014). Furthermore, PCS and Unite Community were instrumental in the production of the Ken Loach film ‘I Daniel Blake’, helping to identify former Jobcentre staff to appear in it (PCS national 5). This award-winning and widely viewed film drew attention to precisely the same issues that campaigners were focused on.

The campaigns in some cases were disruptive. One campaign, Boycott Workfare, used freedom of information act requests to identify local authorities that were using the scheme, then named and shamed municipalities and employers using these placements online. They advised clients not to cooperate with front-line staff sending them to placements, and picketed and leafleted employers using ‘workfare’ placements (presentations in Oxford and Vienna, 2013), and the scheme collapsed in 2015 after several employers withdrew.

Disruption, however, does not equal influence. Campaigners against Atos and the reassessments of disabled people won a victory in the court of public opinion, making the firm’s brand toxic (PCS local 4). Activists were unenthusiastic about the outcome, in part because ‘the only thing that will change is that whichever stupid company that will take it up . . . the assessments will be the same. I think it’s a rebranding exercise’ (Claimants 4).

It was difficult for campaigners to build broad societal solidarity to defend social security. It was not impossible: campaigners embarrassed the government, its contractors and employers enough to disrupt a major workfare scheme and the Atos contract. Evidence on shifts in public opinion towards the benefits system and the unemployed is mixed, with long-term deterioration in the ISSP data (especially pronounced in 2006–2016; see Table 1), but small increases in support for spending on benefits and opposition to cuts in 2011–2017 in the British Social Attitudes Survey (Kelley et al., 2017). The government policy of punishment and restrictions has continued.

## Comparison

The aim of this research was to shed light on the factors that help and hinder the construction of solidarity in the defence of the welfare state. Trade unionists in the NHS fought against privatisation to avoid a degradation of members’ working conditions, to protect local public services and more broadly to uphold the founding principles of the NHS. For PCS in social services, reversing reforms was similarly motivated, not only by the need to protect claimants and welfare services, but also to remove the pressures that assessments and sanctioning had put on the labour process of those working in the sector. This echoes findings on union responses to neoliberal reforms in public services showing that unions often defend simultaneously members’ terms and conditions of employment and benefits and services for their clients (Foster and Scott, 1997; Greer et al., 2013; Jalette and Hebdon, 2012; Teicher et al., 2006). The comparison between health and social services, however, also highlights differences, in particular the factors that help or hinder the building of solidarity. Table 3 summarises the comparison between the cases.

**Table 3.** Comparison of the cases.

	Social security	Healthcare
Campaigners:	PCS, Unite Community, local/national claimant groups	Unite, UNISON, local campaigners
Union resources	High membership density, low fragmentation	Medium member density, high fragmentation
Scale of campaigns	National	Local
Grievances	Tightening of targeting: conditionality, sanctions, degradation of work	Threat to universal service: marketisation, privatisation, degradation of work
Building solidarity:	More difficulty	Less difficulty
Between campaigners	Open conflict	Hidden conflict
In society more broadly	Divided public, some disruption of government initiatives	Much public support, much disruption of government initiatives

Because of differences in tactics, organisation and priorities, building solidarity among campaigners could be a challenge, whether conflicts were hidden (NHS) or in the open (social security). Most groups saw themselves as a ‘sword of justice’ (Flanders, 1970) and framed policy issues in broad social justice terms; this served as a bridge between the different groups to build solidarity in taking collective action (Morgan and Pulignano, 2020). Tensions often stemmed from differences in how to best protect welfare services (tactics) rather than why this was necessary (framing), showing that even when interests overlap and goals are shared, considerable effort is needed in constructing a shared sense of purpose (Gumbrell-McCormick and Hyman, 2015; Lévesque and Murray, 2010; Tassinari and Maccarrone, 2020).

Managing these problems was more difficult in the social security case than in healthcare. This was not because of differences between unions in their power resources or strategic capacities (Lévesque and Murray, 2010): PCS in the social security case benefited from high union membership and a pride in organising, democracy and radical politics (Hodder, 2014), while the NHS unions had lower membership density and a more fragmented structure. Moreover, campaigns to defend social security were national, whereas NHS campaigning remained local, with limited involvement of national unions.

More important for explaining this difference were the macro-level factors of differing welfare architectures motivated by universalism or targeting (Korpi and Palme, 1998). Social security campaigners faced persistent difficulties finding public support to defend jobcentres facing closure or to fight sanctions: cuts to benefits, and not privatisation of services, were at the core of PCS’ coalition work. Public support for government aid to the unemployed remained low during the years of these campaigns, and the labour movement was divided over the sanctions issue. Consequently, much campaigning by PCS and claimants groups focused on changing perceptions. NHS campaigners, by contrast, generally won support from the public. Although the work of campaigning was necessary to raise awareness and explain the intricacies of what was at stake, activists drew on broad cross-class support, including support of the Labour Party in its NHS-centric 2015 general election campaign platform.

The proximate cause of the conflict between claimants groups and trade unionists, however, was not differences in priorities or tactics: instead, it was the labour process of administering targeted welfare benefits. Targeting was carried out using sophisticated top-down management control systems (Carter et al., 2011; Esbenshade et al., 2016; Greer et al., 2018) that forced union members on the front line to assess and sanction claimants in ways that were sometimes traumatising and often generated grievances. These were attributed not only to policymakers and agencies implementing policies, but also to front-line workers themselves. These conflicts on the frontline of service provision had no equivalent in the NHS, where reforms had also affected the labour process of healthcare workers, causing exhaustion and making mobilisation difficult, but without leading patients to question who were allies (‘us’) and who were not (‘them’) (Kelly, 1998).

The active construction of solidarity requires collective identification and attribution of an injustice, a sharing of interests and the feeling of trust and reciprocity (Kelly, 1998; Morgan and Pulignano, 2020; Wills and Simms, 2004). Our argument is that welfare architectures can help or hinder this process. Building solidarity is more difficult when defending targeted benefits (social security) than universal ones (NHS); although social security manages risks for society as a whole, targeting benefits at the poor reinforces stigma of the claimants and the system as a whole. Although NHS campaigners were hampered by low member engagement and a fragmented union landscape, this was compensated by strong public support for public universal healthcare. Social security campaigners, by contrast, even with superior resources and strategic capacities, had difficulty in bringing groups together because of the way welfare reforms caused divisions between unions, claimants groups, political parties and the public. Representations of unemployed welfare claimants added to the difficulty of winning public support, although these too may be shaped by welfare institutions (Larsen and Dejgaard, 2013). Welfare state institutions can thus strengthen and sustain social bonds, but they can also reinforce social divides, producing opportunities and obstacles for campaigners building solidarity.

## Conclusion

At a time of neoliberal reform, defending the welfare state depends on the active construction of solidarity by campaigners. Yet, welfare state institutions shape the conditions in which this solidarity is built. The welfare state employs large numbers of union members who believe in the importance of their work and serves even larger numbers of citizens who depend on benefits and services. Both groups have a material self-interest in fighting cuts and privatisation, and the active construction of solidarity was observed in both cases. As noted above, however, there was a difference: the universalism of the NHS was an important part of the case against privatisation, whereas the targeting of social security led to conflict between trade unionists and claimants and weaker public support for campaigners. This confirms that welfare architectures shape the active construction of solidarity in defence of the welfare state, but also raises new questions about how and why, particularly in the labour process in public services.

Future research will undoubtedly examine how and why campaigners succeed or fail when defending the welfare state. To test the generalisability of this article’s arguments, researchers could ask whether targeting always frustrates the building of solidarity, and whether universalism is always conducive to building solidarity. Can unions and other campaigners develop the strategic capacity needed to overcome social divisions exacerbated by targeting? Other questions are beyond the scope of this article’s analysis. What are the necessary or sufficient conditions for a successful campaign to disrupt the implementation of neoliberal policies or to gain enough influence to propose alternatives? When does occupational solidarity lead to unions to participate in, or lead, broader campaigning rather than abstaining (as with the Royal Colleges)? When is the kind of solidarity strategically built by organisers sufficient, and when do campaigners need ‘spontaneous solidarities’ to win?

## Notes

1. See posts on boycottworkfare.org and johnnyvoid.wordpress.com, both from 23 October 2013.
